# A simple model for viscoelastic crack propagation

**DOI:** 10.1140/epje/s10189-020-00001-w

**Published:** 2021-02-11

**Authors:** B. N. J. Persson

**Affiliations:** PGI-1, FZ Jülich, Jülich, EU Germany

## Abstract

**Abstract:**

When a crack propagates in a viscoelastic solid, energy dissipation can occur very far from the crack tip where the stress field may be very different from the $r^{-1/2}$ singular form expected close to the crack tip. Most theories of crack propagation focus on the near crack tip region. Remarkable, here I show that a simple theory which does not account for the nature of the stress field in the near crack tip region results in a crack propagation energy in semiquantitative agreement with a theory based on the stress field in the near crack tip region. I consider both opening and closing crack propagation and show that for closing crack propagation in viscoelastic solids, some energy dissipation processes must occur in the crack tip process zone. The theory is illustrated by new experimental results for the adhesive interaction between a silica glass ball and a silicone rubber surface.

**Graphic abstract:**

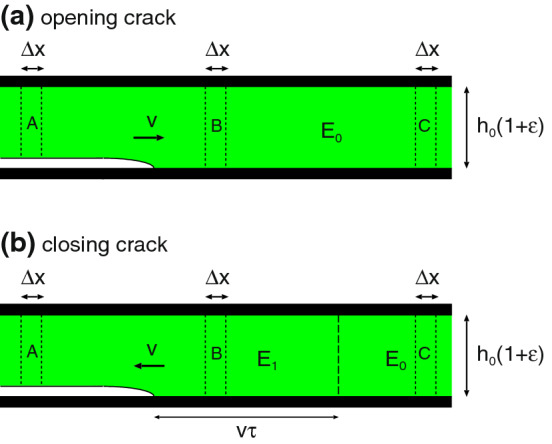

## Introduction

The cohesive strength of solids usually depends on crack-like defects and the energy to propagate cracks in the material. Similarly, the strength of the adhesive bond between two solids is usually determined by the energy to propagate interfacial cracks. Here, we are interested in crack propagation in viscoelastic materials, such as rubber [1–10]. This topic is of great importance, e.g., for adhesion [2], or for the wear of tires or wiper blades, which result from the removal of small rubber particles by crack propagation [11].

When a crack propagates in a viscoelastic solid, energy dissipation can occur very far from the crack tip where the stress field may be very different from the $r^{-1/2}$ singular form expected close to the crack tip. Most theories of crack propagation focus on the near crack tip region, where the stress field takes the $r^{-1/2}$ singular form. Here, I show that neglecting the detailed form of the stress field close to the crack tip results in a crack propagation energy in semiquantitative agreement with a treatment which includes the singular stress field in the near crack tip region.

In Sect. 2, I present a very simple theory for the energy dissipation associated with opening and closing crack propagation in viscoelastic solids. Section 3 presents numerical result. In Sect. 4, I present experimental results for the interaction force between a glass sphere and a silicone rubber slab during approach and retraction and discuss the results in light of the theory. Section 5 contains the summary and conclusions.

## Theory of crack propagation in viscoelastic solids

**Fig. 1:**
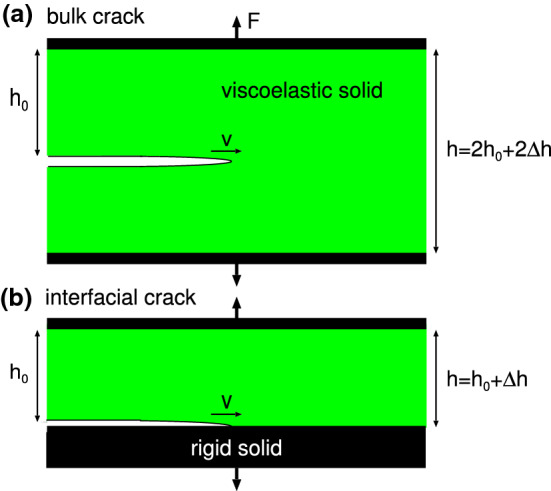
**a** Crack propagation in the bulk of a viscoelastic solid (cohesive crack propagation) and **b** at the interface between a viscoelastic solid and a countersurface (adhesive crack propagation)

A crack in a viscoelastic solid can propagate in the bulk or at an interface. For a bulk crack (Fig. 1a) the stress and strain are usually very high close to the crack tip and nonlinear effects, involving the breaking of strong covalent bonds, chain pullout and cavity formation, will occur close to the crack tip. This region of space is denoted the crack tip process zone, the detailed nature of which is an active research field.

Interfacial crack propagation occurs in many applications, e.g., between rubber materials and a hard countersurface as for pressure sensitive adhesives (Fig. 1b). In this case the strain and stresses at a crack tip can be much smaller, in particular if the interaction at the interface is dominated by the weak van der Waals interaction. In this case nonlinear viscoelastic effects may occur only in a very small region close to the crack tip where the bond breaking occurs. However, for very soft materials, like the weakly cross-linked rubber compounds used in pressure sensitive adhesives, strongly nonlinear effects (such as cavitation and stringing) may occur in a large region close to the crack tip [12, 13].

### Viscoelastic modulus

Assume that a rectangular block of a linear viscoelastic material is exposed to an elongation stress $\sigma (t)$. This will result in a strain $\epsilon (t)$. If we write

1 $$\begin{aligned} \sigma (t)= & {} \int _{-\infty }^\infty \mathrm{d}\omega \ \sigma (\omega ) e^{-i\omega t} \end{aligned}$$

2 $$\begin{aligned} \epsilon (t)= & {} \int _{-\infty }^\infty \mathrm{d}\omega \ \epsilon (\omega ) e^{-i\omega t} \end{aligned}$$

then

3 $$\begin{aligned} \sigma (\omega )=E(\omega ) \epsilon (\omega ) \end{aligned}$$

**Fig. 2:**
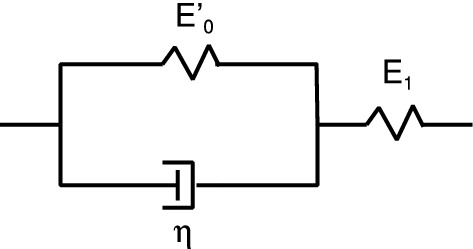
Three-element viscoelastic model used in model calculation of the crack propagation energy *G*(*v*). The low frequency modulus $E(0)=E_0=E_0'E_1/(E_0'+E_1)$ and the high frequency modulus $E(\infty )=E_1$ and the viscosity $\eta$ are indicated

For viscoelastic materials like rubber the viscoelastic modulus $E(\omega )$ is a complex quantity, where the imaginary part is related to energy dissipation (transfer of mechanical energy into the disordered heat motion). In the study below, we will use the three-element rheological model illustrated in Fig. 2. For this model the viscoelastic modulus is

4 $$\begin{aligned} E= {E_0 E_1 (1-i\omega \tau ) \over E_1-i\omega \tau E_0} \end{aligned}$$

**Fig. 3:**
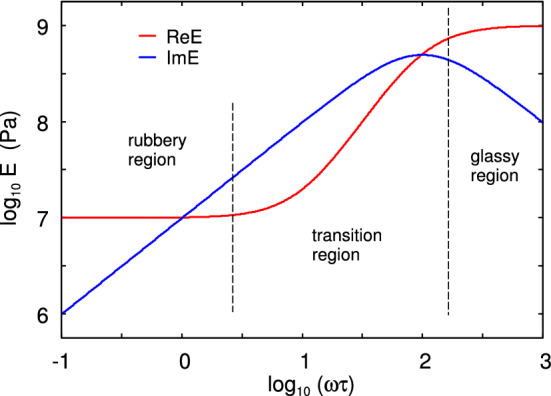
The real and the imaginary part of the viscoelastic modulus as a function of frequency $\omega$ (log–log scale) for the three-element model shown in Fig. 2 with $E_0=10^7\, {\mathrm{Pa}}$ and $E_1=10^9\,{\mathrm{Pa}}$

**Fig. 4:**
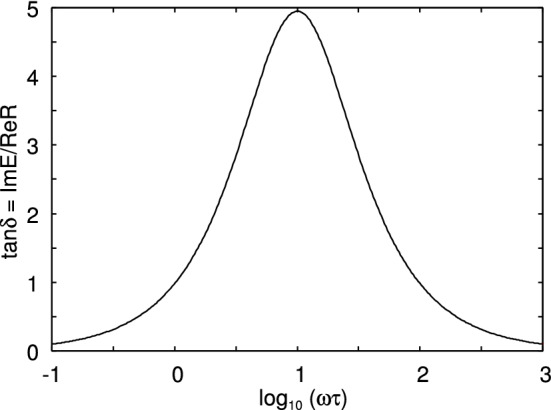
The viscoelastic loss function ${\mathrm{tan}}\delta = {\mathrm{Im}}E/{\mathrm{Re}}E$ as a function of the logarithm of the frequency $\omega$ for the three-element model shown in Fig. 2 with $E_0=10^7\,{\mathrm{Pa}}$ and $E_1=10^9\,{\mathrm{Pa}}$. Note that ${\mathrm{tan}}\delta$ is maximal in the transition region, and this region is most important for many energy loss processes such as rubber friction. However, the viscoelastic crack propagation energy is dominated by energy dissipation processes occurring in the rubbery region

Figure 3 shows the dependency of $E(\omega )$ on frequency (log–log scale), and Fig. 4 shows the loss tangent ${{\mathrm{Im}}}E/{\mathrm{Re}}E$.

For low frequencies (or high temperatures) the rubber responds as a soft elastic body (rubbery region) with a modulus $E(\omega )$ of order $\approx 1 \ {\mathrm{MPa}}$ for the rubber used in tires or $\approx 1 \ {\mathrm{kPa}}$ for the weakly cross-linked rubber used in pressure sensitive adhesive films. At very high frequencies (or low temperatures) is behaves as a stiff elastic solid (glassy region) with the Young’s modulus $E(\omega )$ of order $\approx 1 \ {\mathrm{GPa}}$. In the transition region it exhibits strong internal damping (Fig. 4), and this region is important for many energy loss processes, e.g., rubber friction. Real rubber exhibits a broad distribution of relaxation times, rather than the single relaxation time as in (4), but already the simple three-element model exhibits the basic physics of relevance here.

**Fig. 5:**
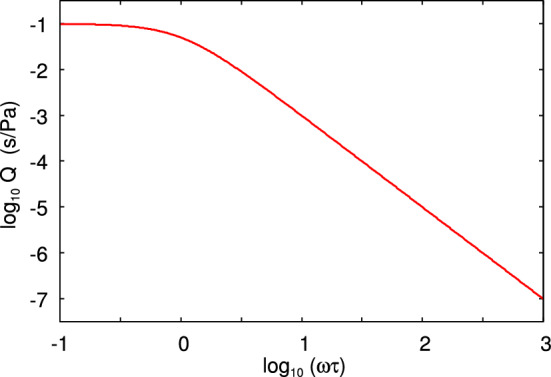
The crack loss function $Q(\omega )=(1/\omega ){\mathrm{Im}}[1/E(\omega )]$ as a function of the frequency $\omega$ (log–log scale) for the three-element model shown in Fig. 2

The viscoelastic modulus $E(\omega )$ is a causal linear response function. This imply that the real and the imaginary part of $E(\omega )$ are not independent functions but given one of them one can calculate the other one using a Kramers–Kronig equation. One can also derive sum rules, and the most important in the present context is

5 $$\begin{aligned} {1 \over E_0} -{1 \over E_1}= {2\over \pi } \int _{0}^\infty \mathrm{d}\omega {1 \over \omega } {\mathrm{Im}} {1\over E( \omega )}, \end{aligned}$$

and

6 $$\begin{aligned} E_0 - E_1= {2\over \pi } \int _{0}^\infty \mathrm{d}\omega {1 \over \omega } {\mathrm{Im}} E( \omega ), \end{aligned}$$

where $E_0=E(0)$ is the static ($\omega =0$) modulus, and $E_1=E(\infty )$ the modulus for infinite high frequency $\omega = \infty$. The function

7 $$\begin{aligned} Q(\omega )={1\over \omega } {\mathrm{Im}} {1\over E(\omega )} \end{aligned}$$

occurring in the integral in (5) is very important in viscoelastic crack propagation, and we denote it as the crack loss function. It is shown in Fig. 5 for the same model rubber as in Fig. 3. Note that $Q(\omega )$ decays monotonically with increasing frequencies and is hence largest in the rubbery region in spite of the small magnitude of the damping in this frequency region.

### Viscoelastic energy dissipation in rectangular strips

The energy dissipated per unit volume when a strip of material is (dynamically) stretched is given by

$$\begin{aligned} U = \int _{-\infty }^\infty \mathrm{d}t \ {{\dot{\epsilon }}} (t) \sigma (t) \end{aligned}$$

Using (1) and (2) and that

$$\begin{aligned} \int _{-\infty }^\infty \mathrm{d}t \ e^{-i(\omega +\omega ') t} = 2 \pi \delta (\omega +\omega ') \end{aligned}$$

we get

8 $$\begin{aligned} U = 2 \pi \int _{-\infty }^\infty \mathrm{d}\omega \ (-i \omega )\epsilon (\omega ) \sigma (-\omega ) \end{aligned}$$

Using $\sigma (\omega ) = E(\omega ) \epsilon (\omega )$ we get

9 $$\begin{aligned} U= & {} 2 \pi \int _{-\infty }^\infty \mathrm{d}\omega \ (-i \omega )\epsilon (\omega ) E(-\omega ) \epsilon (-\omega )\nonumber \\= & {} 4 \pi \int _{0}^\infty \mathrm{d}\omega \ \omega |\epsilon (\omega )|^2 [-{\mathrm{Im}} E(\omega )] \end{aligned}$$

and using $\epsilon (\omega ) = \sigma (\omega )/E(\omega )$ gives

10 $$\begin{aligned} U= & {} 2 \pi \int _{-\infty }^\infty \mathrm{d}\omega \ (-i \omega ){\sigma (\omega ) \over E(\omega )}\sigma (-\omega )\nonumber \\= & {} 4 \pi \int _{0}^\infty \mathrm{d}\omega \ \omega |\sigma (\omega )|^2 {\mathrm{Im}} {1\over E(\omega )} \end{aligned}$$

**Fig. 6:**
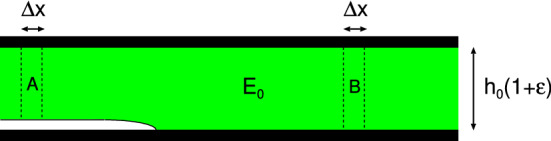
Crack in an elastic solid. For an opening crack the elastic energy stored in the segment B (of width $\Delta x$) is used to break the bonds in a surface area of width $\Delta x$ (transition ${\mathrm{B}} \rightarrow {\mathrm{A}}$). For a closing crack the gain in surface energy when the surfaces close over a region of width $\Delta x$ is used to stretch the strip A (of width $\Delta x$) (transition ${\mathrm{A}} \rightarrow {\mathrm{B}}$)

**Fig. 7:**
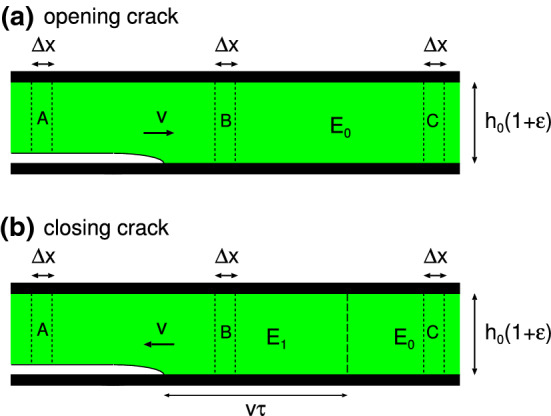
Fast moving opening crack (**a**) and closing crack (**b**) in thin viscoelastic slab under tension. In (**a**) the viscoelastic energy dissipation results in an effective crack propagation energy $G\approx (E_1/E_0)G_0$ which is enhanced by a factor $E_1/E_0$. In (**b**) viscoelastic energy dissipation results in an effective crack propagation energy $G\approx (E_0/E_1)G_0$ which is reduced by a factor $E_0/E_1$ (see text for details)

### Viscoelastic crack: qualitative discussion

When an opening crack propagates in the bulk of a viscoelastic solid, the breaking of the bonds in the crack tip process zone is usually an irreversible process: The broken (dangling) bonds formed during the crack opening react quickly with molecules from the atmosphere or with mobile molecules in the solid. Hence if the external crack driving force is removed, no closing crack propagation involving the reformation of the original bonds will occur. However, for interfacial crack propagation the situation may be very different. Thus, in many cases rubber bind to a countersurface mainly with the weak and long-ranged van der Waals bonds. In this case the bonds broken during crack opening and the bonds formed during crack closing may be very similar, and we will assume this to be the case in what follows.

Consider first a crack in an elastic solid. We consider the setup illustrated in Fig. 6. Both sides of a rectangular slab of an elastic material are bonded to rigid plates. The rigid plates are displaced so that the height of the elastic solid increases from $h_0$ to $h_0+\Delta h= h_0(1+\epsilon _0)$, where the strain $\epsilon _0 = \Delta h/h_0$. Assume now that an interfacial crack occurs and let $\gamma$ be the energy per unit area to break the bonds between then the solids at the lower interface. For a stationary crack energy conservation require that the elastic energy stored in the strip of width $\Delta x$ far in front of the crack tip is equal to the energy to break the bonds at the interface, i.e.,

11 $$\begin{aligned} \gamma \Delta x = {1\over 2} \sigma _0 \epsilon _0 h_0 \Delta x = {1\over 2} h_0 E_0 \epsilon _0^2 \Delta x \end{aligned}$$

For an elastic solid, neglecting emission of elastic waves from the crack tip, (11) is valid both for stationary and moving (opening or closing) cracks.

Consider now a crack in viscoelastic solid. For a stationary crack the condition (11) is still valid, where $E_0$ is the static (or low frequency) modulus. However, stationary cracks are of no real interest as they will not result in failure of the material. For a moving crack in a viscoelastic solid (11) is no longer valid because of viscoelastic energy dissipation. If *P* denotes the viscoelastic energy dissipation per unit time, then when the tip has moved the distance $\Delta x = v \Delta t$ the viscoelastic energy dissipation equal $P\Delta t$. For an opening crack the energy conservation condition becomes

$$\begin{aligned} \gamma \Delta x + P\Delta t= {1\over 2} h_0 E_0 \epsilon _0^2 \Delta x \end{aligned}$$

or

$$\begin{aligned} \gamma v + P_{{\mathrm{open}}}= {1\over 2} h_0 E_0 \epsilon _0^2 v \end{aligned}$$

For a closing crack we get instead

$$\begin{aligned} \gamma v = P_{{\mathrm{close}}}+{1\over 2} h_0 E_0 \epsilon _0^2 v \end{aligned}$$

Physically, for a closing crack the energy gained by the binding of the solids at the crack interface is in part lost as viscoelastic energy dissipation inside the solid, and in part stored as elastic energy in the stretched rubber (far) behind the crack tip.

The energy to propagate the (opening or closing) crack is given by the elastic energy stored far away from the tip and is denoted by *G*:

$$\begin{aligned} G={1\over 2} h_0 E_0 \epsilon _0^2 \end{aligned}$$

Thus, we get

$$\begin{aligned}&G_0 v + P_{{\mathrm{open}}}= G_{{\mathrm{open}}} v\\&G_0 v = P_{{\mathrm{close}}}+G_{{\mathrm{close}}} v \end{aligned}$$

where $$G_0=\gamma $$.

For an opening crack, as the crack speed $v\rightarrow \infty$ we have $G/G_0 \rightarrow E_1/E_0$ but for a closing crack $G/G_0 \rightarrow E_0/E_1$. These results can be understood by considering the simple crack problem shown in Fig. 7.

Figure 7 shows a fast moving opening crack (a) and closing crack (b) in a thin viscoelastic slab under tension. In case (a) the slab is elongated by $h_0 \epsilon _0$, and we wait until a fully relaxed state is formed before inserting the crack. Thus, the elastic energy stored in the strip C of width $\Delta x$ is $\sigma _0 \epsilon _0 h_0 \Delta x/2=E_0 \epsilon _0^2 h_0 \Delta x/2$. This energy is partly used to break the interfacial bonds and partly dissipated due to the material viscoelasticity.

Consider a slab of material of width $\Delta x$ as it moves from one side of the crack to the other side. During this transition it will experience a (elongation) stress $\sigma (t)$ which for a very fast moving crack can be considered as a step function $\sigma = \sigma _0$ for $t<0$ and $\sigma = 0$ for $t>0$, where $t=0$ corresponds to the case where the segment $\Delta x$ is at the crack tip.

The work done by the external force acting on the segment $\Delta x$ must equal the energy which is used to break the bonds in the segment of width $\Delta x$. If we denote this work with $U h_0 \Delta x$, then

$$\begin{aligned} U=-\int _{-\infty }^\infty \mathrm{d}t \ \sigma {{\dot{\epsilon }}} = -\sigma _0 \int _{-\infty }^0 \mathrm{d}t \ {{\dot{\epsilon }}} = \sigma _0 \left[ \epsilon _0-\epsilon (0)\right] \end{aligned}$$

where we have used that $\epsilon =\epsilon _0 = \sigma _0 /E_0$ for $t=-\infty$. Now the strain $\epsilon (0)$ for $t=0$ is actually undefined because the stress make a step-like change at $t=0$. One can show that the correct way to make $\epsilon (0)$ well defined is to use

$$\begin{aligned} \epsilon (0) = {1\over 2} \left[ \epsilon (0^+)+\epsilon (0^-)\right] \end{aligned}$$

where $0^+$ and $0^-$ are infinitesimal positive and negative numbers. Since $\epsilon (0^-)=\epsilon _0$ and

$$\begin{aligned} \epsilon (0^+) = \sigma _0\left( {1\over E_0}-{1\over E_1}\right) \end{aligned}$$

where we have subtracted the instantaneous reduction in the strain due to the instantaneous (high frequency) elastic response (with modulus $E_1=E(\infty )$). Thus, we get

$$\begin{aligned} U= {1\over 2} {\sigma _0^2 \over E_0} -{1\over 2} \sigma _0^2 \left( {1\over E_0}-{1\over E_1}\right) \end{aligned}$$

For a crack in an elastic solid (neglecting energy dissipation from phonon emission from the crack tip) $E_0=E_1$ and we get the standard result that the elastic energy $\sigma _0^2/(2 E_0)$ can be fully used to break the bonds at the crack tip, but in the present case (for a fast moving crack)

$$\begin{aligned} U={1\over 2} {\sigma _0^2 \over E_1} \end{aligned}$$

and the condition $U h_0 \Delta x = G_0 \Delta x$ gives

$$\begin{aligned} {1\over 2} h_0 {\sigma _0^2 \over E_0} {E_0\over E_1} = G_0 \end{aligned}$$

or $$G=G_0 E_1/E_0$$.

For the closing crack (case (b)) the situation is different: For a fast moving crack the strip A is quickly elongated when it approaches the crack tip, which requires a large stress $\sigma =E_1 \epsilon$ determined by the high frequency modulus $E_1$. Since the crack moves very fast, the stress in the strip will remain at this large value even when the crack tip has moves far away from the strip as in position B. However, due to viscoelastic relaxation the stress will finally arrive at the relaxed value $\sigma =E_0 \epsilon$ as at position C. The time this takes depends on the nature of the viscoelastic relaxation process, e.g., for a process characterized by a single relaxation time $\tau$, a time $t> \tau$ (and distance $s>v\tau$) would be needed to reach the relaxed state. During this relaxation mechanical energy is converted into heat. Since the crack tip is far away from the region where this relaxation process takes place, it does not know about it, and the interfacial binding energy is converted into elastic energy in the rapid stretching of the strip in the process going from strip position A to B. Thus, $G_0 \Delta x = E_1 \epsilon _0^2 h_0 \Delta x/2$. However, the crack propagation energy *G* refer to the relaxed state configuration so that $G \Delta x = E_0 \epsilon _0^2 h_0 \Delta x/2$. Thus, $G = E_0 \epsilon _0^2 h_0/2 = (E_0/E_1) E_1 \epsilon _0^2 h_0/2 = (E_0/E_1)G_0$.

This result for a closing crack tip can also be derived using the same approach as used for the opening crack. Thus in the present case, for a fast moving closing crack the strain rather than the stress is known: $\epsilon (t)=0$ for $t<0$ and $\epsilon (t)=\epsilon _0$ for $t>0$. Thus, ${{\dot{\epsilon }}} (t) = \epsilon _0 \delta (t)$ and

$$\begin{aligned} U=-\int _{-\infty }^\infty \mathrm{d}t \ \sigma {{\dot{\epsilon }}} = -\epsilon _0 \sigma (0) = -\epsilon _0 {1\over 2} \left( \sigma (0^+)+\sigma (0^-) \right) \end{aligned}$$

Since $\sigma (0^-) = 0$ and $\sigma (0^+) = E_1 \epsilon _0$ we get

$$\begin{aligned} U=-{1\over 2} {E_1 \epsilon _0^2} \end{aligned}$$

and the condition $U h_0 \Delta x + G_0 \Delta x=0$ gives

$$\begin{aligned} {1\over 2} h_0 {E_1 \epsilon _0^2} = {1\over 2} h_0 {\sigma _0^2 \over E_0} {E_1\over E_0} = G_0 \end{aligned}$$

or $$G=G_0 E_0/E_1$$.

### Opening crack

The discussion in Sect. 2.3 can be easily generalized to a crack moving at a finite speed in a viscoelastic solid. As a strip $\Delta x$ of material moves through the crack tip region, for an opening crack we assume it experiences the stress

$$\begin{aligned} \sigma (t)= & {} \sigma _0 \ \ \ {\mathrm{for}} \ \ \ t<-\tau ^* \\ \sigma (t)= & {} \sigma _0 {\tau ^* -t \over 2 \tau ^*} \ \ \ {\mathrm{for}} \ \ \ -\tau ^*<t<\tau ^* \\ \sigma (t)= & {} 0 \ \ \ {\mathrm{for}} \ \ \ t> \tau ^* \end{aligned}$$

where $v \tau ^* = a$ is the width of the crack tip process zone. We get

12 $$\begin{aligned} \sigma (\omega ) = {1\over 2 \pi } \int _{-\infty }^\infty \mathrm{d}t \ \sigma (t) e^{i\omega t} = {i \tau ^* \sigma _0 \over 2 \pi } {{\mathrm{sin}} \xi \over (i0^+ - \xi )\xi }\quad \end{aligned}$$

where $\xi = \omega \tau ^*$ and where $0^+$ is an infinitesimal positive number. Substituting (12) in (10) and using $\sigma _0 = E_0 \epsilon _0$ gives

13 $$\begin{aligned} U_{\mathrm{open}} = (\epsilon _0 E_0 )^2 {1\over \pi } \int _0^\infty \mathrm{d}\omega \ R(\omega ) {1\over \omega } {\mathrm{Im}} {1\over E(\omega )} \end{aligned}$$

where

14 $$\begin{aligned} R(\omega ) = \left( {\mathrm{sin} (\omega \tau ^*)\over \omega \tau ^*}\right) ^2 \end{aligned}$$

We expect $v \tau ^* \approx a$, where *a* is the crack tip radius. In Ref. [7] we used a different approach where $R(\omega )$ was replaced by

$$\begin{aligned} F(\omega )=\left[ 1-\left( { \omega \over \omega _{\mathrm{c}}}\right) ^2\right] ^{1/2} \end{aligned}$$

where $\omega _{\mathrm{c}} = 2\pi v/a$. Note that if we expand *R* and *F* to quadratic order in $\omega$, then the two expressions agree if we choose $\tau ^* = (3/2)^{1/2} a/(2 \pi v)$.

Energy conservation gives

$$\begin{aligned} {1\over 2} h_0 \Delta x E_0 \epsilon _0^2 = h_0 \Delta x U_{\mathrm{open}}+ \gamma \Delta x \end{aligned}$$

or using (13),

$$\begin{aligned} {1\over 2} h_0 E_0 \epsilon _0^2 \left( 1- E_0 {2\over \pi } \int _0^\infty \mathrm{d}\omega \ R(\omega ) {1\over \omega } {\mathrm{Im}} {1\over E(\omega )} \right) =\gamma \end{aligned}$$

Since $\gamma = G_0$ and $G=h_0 E_0 \epsilon _0^2/2$ we get

15 $$\begin{aligned} G={G_0 \over 1- E_0 {2\over \pi } \int _0^\infty \mathrm{d}\omega \ R(\omega ) {1\over \omega } {\mathrm{Im}} {1\over E(\omega )}} \end{aligned}$$

Note that when $v\rightarrow \infty$ we have $\tau ^* \rightarrow 0$ and hence $R\rightarrow 1$. Thus, for very high opening crack speeds

$$\begin{aligned} G (v=\infty )={G_0 \over 1- E_0 {2\over \pi } \int _0^\infty \mathrm{d}\omega \ {1\over \omega } {\mathrm{Im}} {1\over E(\omega )}} \end{aligned}$$

Using (5) this gives $G (v=\infty )=(E_1/E_0) G_0$.

Using (5) we can write (15) as

16 $$\begin{aligned} {G_0 \over G} =1- {E_1 {2\over \pi } \int _0^\infty \mathrm{d}\omega \ R(\omega ) {1\over \omega } {\mathrm{Im}} {1\over E(\omega )} \over 1+ E_1 {2\over \pi } \int _0^\infty \mathrm{d}\omega \ {1\over \omega } {\mathrm{Im}} {1\over E(\omega )}} \end{aligned}$$

which is convenient for numerical calculations.

Note that *G* depends on the crack tip size parameter *a*. Experiments have shown that the crack tip radius increases with the crack tip speed. We can choose *a* so that the stress for $r=a$ is of order characteristic yield stress $\sigma _{\mathrm{c}}$, e.g., the stress to break bonds, which could be strong covalent bonds for cohesive crack propagation. The stress close to the crack tip is given by

$$\begin{aligned} \sigma \approx {C \over r^{1/2}} \end{aligned}$$

At a distance $\sim h_0$ from the crack tip the stress is of order $\sigma _0$ so we expect $C / (\alpha h_0)^{1/2} \approx \sigma _0$, where $\alpha$ is a number of order unity. Thus, the stress at the crack tip $r=a$ is $\sigma = \sigma _{\mathrm{c}} \approx \sigma _0 (\alpha h_0/a)^{1/2}$, or using $G= h_0 E_0 \epsilon _0^2/2 = h_0 \sigma _0^2/(2E_0)$ we get

17 $$\begin{aligned} a = {E_0 2 \alpha G\over \sigma _{\mathrm{c}}^2}. \end{aligned}$$

If we choose $\alpha = 1/(4 \pi )$ we obtain the equation derived in Ref. [7]. The tip radius *a*(*v*) depend on the crack tip speed *v*, and using that $G(\infty )=G_0 E_1/E_0$ we get $a(\infty ) = a_0 E_1/E_0$ where $a_0 = a(0)$ is the crack tip radius for very low crack tip speed.

### Closing crack

The strain

$$\begin{aligned} \epsilon (t)= & {} 0 \ \ \ \mathrm{for} \ \ \ t<-\tau ^* \\ \epsilon (t)= & {} \epsilon _0 {t+\tau ^* \over 2 \tau ^*} \ \ \ \mathrm{for} \ \ \ -\tau ^*<t<\tau ^* \\ \epsilon (t)= & {} \epsilon _0 \ \ \ \mathrm{for} \ \ \ t> \tau ^* \end{aligned}$$

We get

18 $$\begin{aligned} \epsilon (\omega ) = {1\over 2 \pi } \int _{-\infty }^\infty \mathrm{d}t \ \epsilon (t) e^{i\omega t} = {i \tau ^* \epsilon _0 \over 2 \pi } {\mathrm{sin} \xi \over (i0^+ +\xi )\xi } \end{aligned}$$

where $\xi = \omega \tau ^*$ and where $0^+$ is an infinitesimal positive number. Substituting (18) in (9) gives

19 $$\begin{aligned} U_{\mathrm{close}} = \epsilon _0^2 {1\over \pi } \int _0^\infty \mathrm{d}\omega \ R(\omega ) {1\over \omega } {\mathrm{Im}} [-E(\omega )] \end{aligned}$$

For closing cracks the energy conservation condition gives

$$\begin{aligned} \gamma \Delta x = {1\over 2} h_0 \Delta x E_0 \epsilon _0^2 + h_0 \Delta x U_{\mathrm{close}} \end{aligned}$$

or using (19),

20 $$\begin{aligned} \gamma = {1\over 2} h_0 E_0 \epsilon _0^2 \left( 1+E_0^{-1} {2\over \pi } \int _0^\infty \mathrm{d}\omega \ R(\omega ) {1\over \omega } {\mathrm{Im}} [-E(\omega )]\right) \nonumber \\ \end{aligned}$$

or

$$\begin{aligned} G={G_0 \over 1+E_0^{-1} {2\over \pi } \int _0^\infty \mathrm{d}\omega \ R(\omega ) {1\over \omega } {\mathrm{Im}} [-E(\omega )]} \end{aligned}$$

Note that when $v\rightarrow \infty$ we have $\tau ^* \rightarrow 0$ and hence $R\rightarrow 1$. Thus, for very high opening crack speeds

$$\begin{aligned} G(v=\infty )={G_0 \over 1+E_0^{-1} {2\over \pi } \int _0^\infty \mathrm{d}\omega \ {1\over \omega } {\mathrm{Im}} [-E(\omega )]} \end{aligned}$$

Using (6) this gives $G (v=\infty )=(E_0/E_1) G_0$. Using (6) we can write (20) as

21 $$\begin{aligned} {G_0 \over G} = 1+{E_1^{-1} {2\over \pi } \int _0^\infty \mathrm{d}\omega \ R(\omega ) {1\over \omega } {\mathrm{Im}} [-E(\omega )] \over 1- E_1^{-1} {2\over \pi } \int _0^\infty \mathrm{d}\omega \ {1\over \omega } {\mathrm{Im}} [-E(\omega )] } \end{aligned}$$

For the crack opening we determined the crack tip radius *a* such as the stress at the crack tip equal the critical stress necessary for bond breaking. This resulted in a radius which, in agreement with experiments, increases with increasing crack tip speed and in particular $a\rightarrow a_0 E_1/E_0$ as the crack tip velocity $v \rightarrow \infty$. However, making the same assumption for the closing crack results in unphysical results, namely $a\rightarrow a_0 E_0/E_1$. We expect $a_0$ to be of order 1 nm so in a typical case with $E_0/E_1 \approx 10^{-3}$ the crack tip radius $a\rightarrow 0.001 \times a_0= 0.01 \ \mathrm{nm}$. But this result is unphysical; the radius cannot be smaller than an atomic length and in fact we expect $a\approx a_0$ for all velocities for a closing crack.

Now, if we choose $a=a_0$ for all crack tip velocities for the closing crack, then the stress $\sigma _1$ at the crack tip for high crack tip speed would be much smaller than the adhesive bonding stress at the crack tip. This imply that large forces will act on the rubber segments at the crack tip and the rubber segments will accelerate and snap into contact, and perhaps undergoes some other rapid event where energy is lost (converted into heat). In fact, Carbone et al have suggested that some slip will occur in the crack tip process zone during contact formation for soft adhesive films. This will make $G_0$ smaller than in the adiabatic limit (since for a closing crack $G_0(v)=\gamma -w$ is the binding energy per unit surface energy, $\gamma$, minus the energy *w* dissipated in the crack tip process zone). The combination (for high crack tip speed) of the viscoelastic reduction factor $E_0/E_1$, and the reduction in $G_0$ with increasing crack tip speed will make adhesion nearly absent during contact formation in typical cases. This is in accordance with experiments where for macroscopic solids, even for solids with very smooth surfaces, in most cases no adhesion can be observed during contact formation (see Sect. 4).

Here we note that the problem addressed above, involving how to determine the crack tip radius *a*, and the related stress mismatch problem, also occur in a modified form in the Barenblatt process zone treatment of the closing crack problem. Thus, for a fast moving crack a region of compressible stress occurs close to the crack tip for which no physical explanation exists [5, 10].

We note that the standard Barenblatt process zone treatment corresponds to a crack moving with a constant speed, and in this model there is no events taking place with a speed unrelated to the crack tip speed. Thus, the rapid flipping (or local stick slip) which may occur in the process zone is absent in this treatment. Still, the repulsive dip (or compression stress) observed for this model for a closing crack may be a manifestation of the fact that something is missing in the Barenblatt process zone model for a closing crack.

**Fig. 8:**
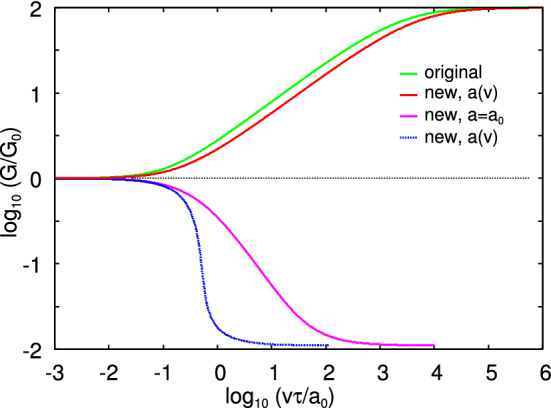
The crack propagation energy as a function of the crack tip speed (log–log scale) using the rheological model defined by (1). The green line is for an opening crack using the original Persson–Brener theory (Ref. [7]) where the viscoelastic energy dissipation is calculated from the crack tip stress field. This result is virtually identical to the result obtained using the Barenblatt process cone model [10]. The red line is the result for an opening crack using the simplified treatment, where the viscoelastic energy dissipation is estimated (from (16) and (17)) using the stretching-segment model (see Sect. 3). The dashed blue line is the (unphysical) result obtained from (21) and (17), where the crack tip radius becomes unphysical small for high crack tip speed ($a\rightarrow (E_0/E_1)a_0$ as $v\rightarrow \infty$). The pink line is the result for closing crack using (21) and assuming a constant crack tip radius $a=a_0$

## Numerical results

We have calculated the crack propagation energy *G*(*v*) using the simple three-element rheology model shown in Fig. 2. In Fig. 8 we show the crack propagation energy as a function of the crack tip speed (log–log scale) using the rheological model defined by (1). The green line is for an opening crack using the original theory (Ref. [7]) where the viscoelastic energy dissipation is calculated from the crack tip stress field. This result is virtually identical to the result obtained using the Barenblatt process cone model [10]. The red line is the result for an opening crack using the simplified treatment where the viscoelastic energy dissipation is estimated (from (16) and (17)) using the stretching-segment picture. The dashed blue line is the (unphysical) result obtained from (21) and (17), where the crack tip radius becomes unphysical small for high crack tip speed ($a\rightarrow (E_0/E_1)a_0$ as $v\rightarrow \infty$). The pink line is the result for closing crack using (21) and assuming a constant crack tip radius $a=a_0$. Comparing the pink line with the red (and green) lines we conclude that if we write for crack opening $G=G_0 (1+f(v))$, then for small $v\tau /a_0$ we have for crack closing $G \approx G_0 /(1+f(v))$. In the Barenblatt crack zone treatment this relation is found to hold approximately for all crack tip velocities [10].

**Fig. 9:**
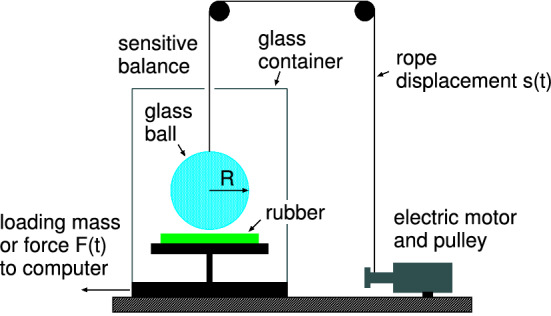
The experimental setup for measuring adhesion

**Fig. 10:**
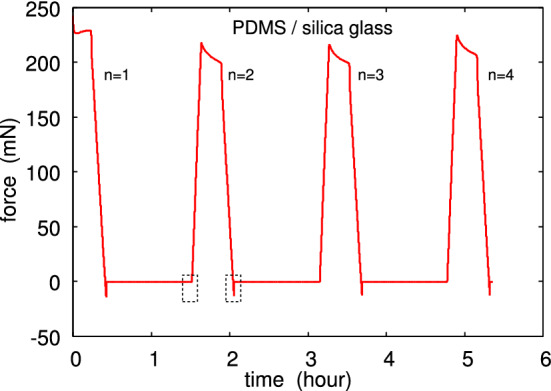
The interaction force between a glass ball (diameter $2R=4 \ \mathrm{cm}$) moved in repeated contact and a flat PDMS surface. The approach and retraction speed is $v_z=0.33\,\upmu {\mathrm{m/s}}$. The dashed rectangular regions are shown magnified in Fig. 11 (crack opening) and Fig. 12 (closing crack)

**Fig. 11:**
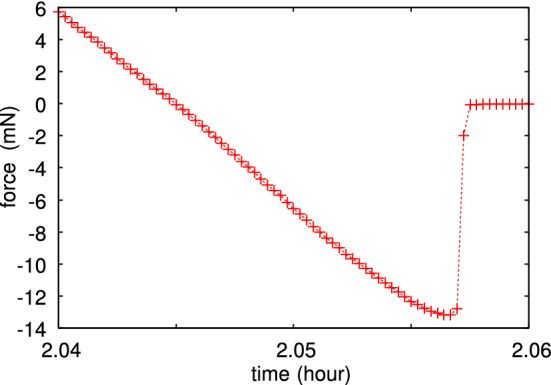
Magnified view of the second pulloff event in Fig. 10. The (radial) crack tip speed just before snap-off is $v_r \approx 14\, \upmu {\mathrm{m/s}}$. The pulloff force corresponds to the work of adhesion $G\approx 0.14 \ {\mathrm{J/m}}^2$. There is one data point per second

**Fig. 12:**
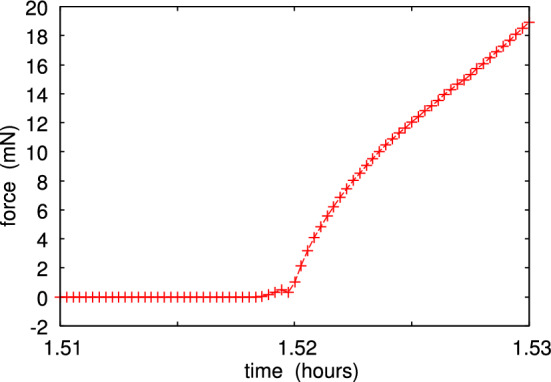
Magnified view of the second contact formation event in Fig. 10. Note the strong adhesion hysteresis: No adhesion is observed during approach, but adhesion is observed during pulloff (Fig. 11). There is one data point per second

**Fig. 13:**
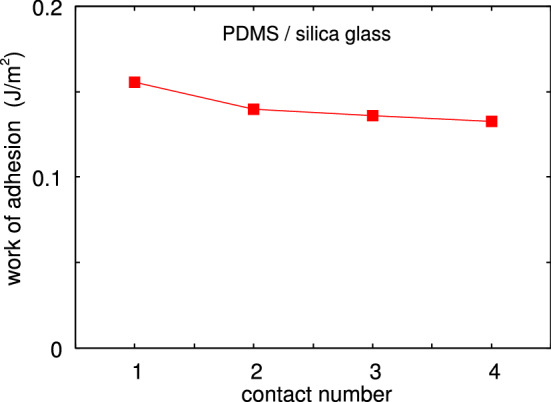
The work of adhesion during pulloff as a function of the contact number. The decrease in the work of adhesion is due to transfer of molecules from the PDMS to the glass ball, passivating the glass surface

**Fig. 14:**
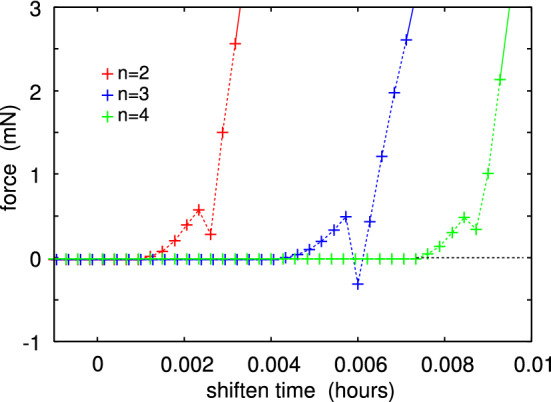
The interaction force between the glass ball and the PDMS surface on approach for the contacts $n=2$, 3 and 4 in Fig. 10. Note the small repulsive barrier before contact which we attribute to the influence of dust particles in the nominal contact region

## Experimental results and discussion

For a very slowly moving adhesive crack the elastic stress at the crack tip must be just balancing the adhesive stress, so no rapid non-thermal instabilities, such as snap-off or snap-in, can occur. We have argued above that for a fast moving closing adhesive crack the crack propagation energy *G*(*v*) must be reduced not only by the viscoelastic factor $E_0/E_1$ but also $G_0(v)$ must be strongly reduced due to rapid events in the crack tip process zone caused by the stress mismatch at the crack tip. This conclusion is supported by adhesion experiments. Thus, adhesion is usually not observed when two macroscopic solids approach each other, while for elastically soft solids strong adhesion may be observed upon separation.

To illustrate this we have studied the adhesion interaction between spherical silica glass balls and rubber. In the experiments we bring a glass ball with diameter $2R=4 \ \mathrm{cm}$ into contact with a rubber substrate as shown in Fig. 9. It is positioned on a very accurate balance (analytic balance produced by Mettler Toledo, model MS104TS/00) which has a reproducibility of $0.01 \ \mathrm{mg}$ (or $\approx 0.1 \ \mu \mathrm{N}$). After zeroing the scale of the instrument we can measure the force on the substrate as a function of time which is directly transferred to a computer at a rate of 1 measurement point per second.

To move the glass ball up and down we have used an electric motor coiling up a nylon cord, which is attached to the glass ball. The pulling velocity as a function of time can be specified on a computer. In the experiments reported on below the glass ball is repeatedly moved up and down, for several contact cycles.

As an example, in Fig. 10 we show the interaction force between the glass ball and a flat polydimethylsiloxane (PDMS) rubber surface, both with very smooth surfaces. The ball moves up and down with the speed $0.33 \ \mu$m/s and we show the interaction force for 4 contacts. Note that no attraction is detected during contact formation, but during pulloff adhesion manifest itself as a negative interaction force. This is illustrated in detail (for the second contact cycle) in Fig. 11 (pulloff) and Fig. 12 (contact formation). The work of adhesion during pulloff as a function of the contact number is shown in Fig. 13. The decrease in the work of adhesion with increasing number of contacts is due to transfer of molecules from the PDMS to the glass ball, passivating the glass surface.

For the second pulloff the work of adhesion $G\approx 0.14 \ {\mathrm{J/m}}^2$. This is a factor of $\sim 2$ larger than the adiabatic work of adhesion between PDMS and a glass surface, which is about $G_0 \approx 0.06 \ {\mathrm{J/m}}^2$. This imply that the viscoelastic enhancement factor $1+f(v,T)$ is a factor of $\approx 2$ (or less), in agreement with calculations [14]. Thus if viscoelasticity would be the only energy dissipation process, we would expect a work of adhesion during contact formation to be $G_{\mathrm{close}} \approx G_0/(1+f(v,T)) \approx 0.03 \ {\mathrm{J/m}}^2$, corresponding to an attractive ball–PDMS force of $\approx 3 \ {\mathrm{mN}}$. However, the small dip in the measured contact formation force is between $0.1-0.8 \ \mathrm{mN}$ (Fig. 14). This imply that some energy dissipation process (with the dissipated energy per unit surface area *w*) must occur in the crack tip process zone during crack closing. In this case part of the energy (per unit surface area) $\gamma$ gained in the bond formation process is lost in the crack tip process zone and $G_0 = \gamma - w < \gamma$. The exact processes occurring is not known but may involve some snap-in or local slip at the contacting interface.

Before the small adhesive dip in the time–force curves in Fig. 14, the ball–flat interaction is repulsive. There are at least two possible origins of this repulsion. One effect is squeeze film: When the ball is very close to the PDMS surface, a hydrodynamic pressure builds up in the air film between the ball and the flat PDMS surface. This force can be estimated using the Navier Stokes equations of fluid dynamics (on the simplified Reynolds equation form). Thus, if *h*(*t*) denotes the shortest ball–flat separation, then for $h<<R$ (see Ref. [15])

$$\begin{aligned} F = 6 \pi \mu R^2 {\dot{h} \over h} \end{aligned}$$

This equation gives a similar dependency on the separation *h* as shown in Fig. 14, but using the viscosity of air ($\mu \approx 1.8\times 10^{-5} \ \mathrm{Pas}$) the magnitude of the calculated force is a factor of $\sim 1000$ too small. Another explanation is that there are one or several dust particles adsorbed on the PDMS surface, which need to be squeezed into the rubber surface (elastic deformation) before the glass–PDMS contact can occur. Since the experiments was performed in the normal atmosphere this is a likely explanation.

When an opening crack propagates in the bulk of an elastomer (cohesive crack), strong covalent bonds are broken at the crack tip. In a recent study [16] using fluorogenic mechanochemistry with quantitative confocal microscopy mapping, it was found how many and where covalent bonds are broken. The measurements reveal that bond scission near the crack plane can be delocalized over up to hundreds of micrometers and increase $G_0$ by a factor of $\approx 100$ depending on temperature and stretch rate, pointing to an intricate coupling between strain rate dependent viscous dissipation and strain dependent irreversible net work scission. These findings shows that energy dissipated by covalent bond scission accounts for a much larger fraction of the total fracture energy than previously believed.

The study above does not give any dependency of the crack propagation energy on the height $h_0$ of the viscoelastic slab. That is, the crack propagation factor $G/G_0 = 1+f(v,T)$ does not depend on the height $h_0$ of the rubber sample assuming the length (in the *x*-direction) *L* of the sample is infinite. This differs from the conclusion derived in Ref. [17] and the proposal by de Gennes [6] that the origin of instabilities in the pulloff of adhesive tape may be due the influence of the finite film thickness on the viscoelastic energy dissipation. However, the present study does not include the singular part of the crack tip stress field, and more studies are needed to understand the role of finite-size effects on the viscoelastic contribution to the crack propagation energy. Furthermore, slabs or films always have a finite extent in the lateral direction which will introduce a finite-size effect even if the results would be independent of the film thickness.

## Summary and conclusion

I have studied crack propagation in a stretched rectangular strip of a viscoelastic solid. I have shown that for an opening crack using a very simple model for the stress field, which describes the stress correctly far from the crack tip but not close to it, gives a viscoelastic crack propagation energy factor $G/G_0=1+f(v,T)$ very close to the one obtained using the Barenblatt process zone model, or the Persson–Brener crack tip model. For a closing crack tip the same approach gives physical reasonable result only if one assumes a constant crack tip radius and assumes that the crack tip process zone energy $G_0$ decreases with increasing crack tip speed, which imply that for high enough crack tip speed dissipate processes, e.g., involving rapid flipping of molecular segment or local slip, occur at the crack tip during closing, which consumes a large fraction of the gain in energy due to the bond formation.
